# Impact of attenuation correction on clinical [^18^F]FDG brain PET in combined PET/MRI

**DOI:** 10.1186/s13550-016-0200-0

**Published:** 2016-06-03

**Authors:** P. Werner, M. Rullmann, A. Bresch, S. Tiepolt, T. Jochimsen, D. Lobsien, M. L. Schroeter, O. Sabri, H. Barthel

**Affiliations:** Department of Nuclear Medicine, Leipzig University Hospital, Leipzig, Germany; Department of Neuroradiology, Leipzig University Hospital, Leipzig, Germany; Day Clinic for Cognitive Neurology, Leipzig University Hospital and Max Planck Institute for Human Cognitive and Brain Sciences, Leipzig, Germany

**Keywords:** PET/MR, Attenuation correction, FDG imaging

## Abstract

**Background:**

In PET/MRI, linear photon attenuation coefficients for attenuation correction (AC) cannot be directly derived, and cortical bone is, so far, usually not considered. This results in an underestimation of the average PET signal in PET/MRI. Recently introduced MR-AC methods predicting bone information from anatomic MRI or proton density-weighted zero-time imaging may solve this problem in the future. However, there is an ongoing debate if the current error is acceptable for clinical use and/or research.

**Methods:**

We examined this feature for [^18^F] fluorodeoxyglucose (FDG) brain PET in 13 patients with clinical signs of dementia or movement disorders who subsequently underwent PET/CT and PET/MRI on the same day. Multiple MR-AC approaches including a CT-derived AC were applied.

**Results:**

The resulting PET data was compared to the CT-derived standard regarding the quantification error and its clinical impact. On a quantitative level, −11.9 to +2 % deviations from the CT-AC standard were found. These deviations, however, did not translate into a systematic diagnostic error. This, as overall patterns of hypometabolism (which are decisive for clinical diagnostics), remained largely unchanged.

**Conclusions:**

Despite a quantitative error by the omission of bone in MR-AC, clinical quality of brain [^18^F]FDG is not relevantly affected. Thus, brain [^18^F]FDG PET can already, even now with suboptimal MR-AC, be utilized for clinical routine purposes, even though the MR-AC warrants improvement.

## Background

No differences in diagnostic quality of the MR component in hybrid PET/MR systems as compared to stand-alone MR systems have been reported [1, 2]. For the PET component, however, attenuation correction (AC) systematically differs from that in PET/CT or stand-alone PET as in PET/MRI the linear photon attenuation coefficients cannot directly be derived. Hence, the standard segmentation-based AC currently provided by the vendors assigns defined attenuation coefficients to different tissue classes (usually fat, soft tissue, air) segmented from a 3D T1-weighted volumetric interpolated breath-hold examination (VIBE) Dixon sequence. In brain imaging, sequential PET/CT vs. PET/MRI cross-evaluation studies reported an underestimation of the average PET signal in PET/MRI due to the omission of cortical bone for several tracers, ranging from 11 to 12 % [3, 4] up to 19 to 25 % [5, 6]. There is an ongoing debate on whether this error is acceptable for clinical use and/or research. Moreover, it was recently argued that this problem is potentially solved with recent AC methods predicting bone information from anatomic MRI or proton density-weighted zero-time imaging [7]. Applying the current standard AC methods, it could be hypothesized that, for brain [^18^F] fluorodeoxyglucose (FDG) PET, cortical hypometabolism may be overestimated in PET/MRI, a drawback which may result in false-positive findings [8]. To test this hypothesis, we examined 13 patients with clinical signs of dementia or movement disorders who subsequently underwent FDG PET/CT and PET/MRI. PET data from PET/MRI were reconstructed using (1) segmentation-based attenuation maps, (2) continuous *μ*-maps derived from the CT, and (3) continuous *μ*-maps predicted from high-resolution anatomical MRI. The respective PET data derived from the outlined reconstruction methods were compared with the gold standard, the PET/CT data, regarding the quantification error and its clinical impact.

## Methods

Thirteen patients with clinical evidence of either dementia or movement disorders underwent brain PET/MRI and PET/CT of random sequence (*n* = 7 PET/MRI-first and *n* = 6 PET/CT-first). Average time from injection to PET/CT and PET/MRI did not differ between the PET/MRI and PET/CT first groups (85 ± 51 vs. 68 ± 38 min, *p* = 0.48, two-tailed *t* test, Table 1). The groups did not differ in age (59 ± 17 years for PET/CT-first and 69 ± 9 years for PET/MRI-first, *p* = 0.2, two-tailed *t* test, Table 1). After injection of 242 ± 39 MBq FDG, the patients were placed in a dimly lit and sound-shielded room to minimize sensory stimulation for at least 30 min before they underwent first imaging. Without a break, the patients were then transferred to the other imaging modality to undergo the second brain scan.

**Table 1:** Patient characteristics

Patient	Age	Gender	Activity	PET/CT	PET/MRI	Imaging diagnosis
	[years]		[MBq]	[min p.i.]	[min p.i.]	
1	70	M	245	38	98	Supranuclear palsy
2	68	F	221	127	35	Unremarkable
3	57	F	215	140	40	Posterior cortical atrophy DD Alzheimer’s dementia
4	73	F	241	164	35	Unremarkable
5	53	M	248	30	73	Unremarkable
6	74	F	220	119	31	Unremarkable
7	72	F	222	55	85	Unremarkable
8	27	M	217	30	78	Unremarkable
9	79	M	312	134	44	Alzheimer’s dementia
10	60	M	210	131	30	Frontotemporal lobar degeneration
11	65	F	258	31	84	Corticobasal degeneration
12	65	F	211	30	86	Unremarkable
13	67	M	334	69	163	Unremarkable

### PET/MRI

Simultaneous brain PET/MR acquisition was performed using an integrated PET/MRI system (Siemens mMR Biograph, Erlangen, Germany, software version VB18P). Patients were positioned in a dedicated PET/MRI head coil. Dynamic brain PET data were acquired in 3D list-mode over 20 min. During PET acquisition, a two-point MRI Dixon sequence (matrix 128 × 192, 126 slices, isotropic voxels 2.6 × 2.6 × 2.6 mm^3^) was acquired. For attenuation correction, attenuation coefficient maps (air, soft tissue, fat) were segmented from the fat, and water images generated by the Dixon sequence and PET_Dixon_ were reconstructed using the built-in OSEM algorithm with a zoom factor of 2.8, eight iterations, 21 subsets, and a 3-mm Gaussian filter (256 × 256 matrix, 127 slices, voxel size 2.8 × 2.8 × 2.03 mm^3^). Apart from diagnostic MR sequences, as they were required according to the clinical question, T1 magnetization-prepared rapid acquisition gradient echo (MPRAGE) data were acquired in all patients (TE = 2.53 ms, TR = 1900 ms, matrix 512 × 512, 176 slices, voxel size 0.48 × 0.48 × 1 mm^3^). In six patients, an additional ultrashort echo time (UTE) sequence was acquired for *μ*-map creation with bone information (TE = 0.07 and 2.46 ms, TR = 11.9 ms, 192 × 192 × 192 voxels, voxel size 1.6 × 1.6 × 1.6 mm^3^).

### PET/CT

**Fig. 1:**
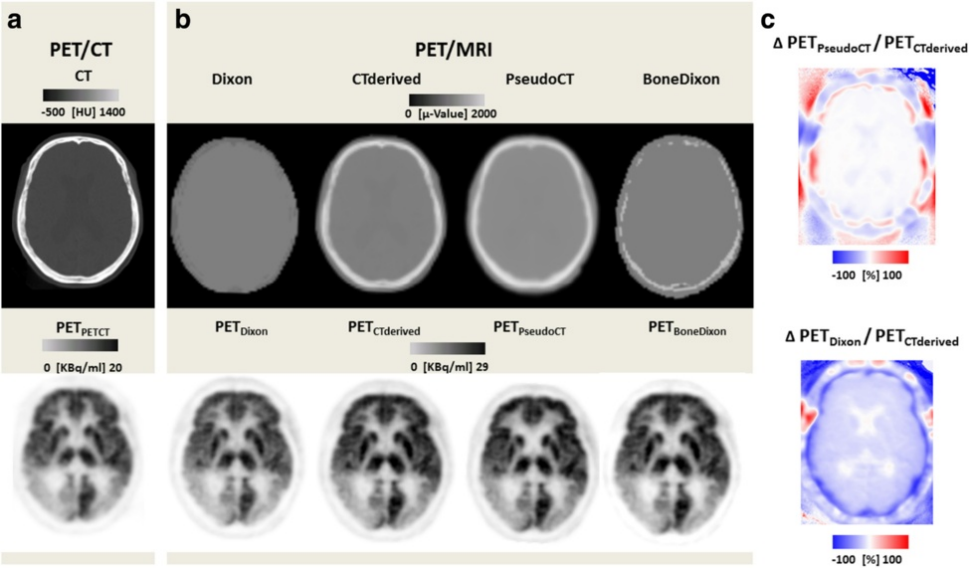
FDG brain PET image reconstruction and postprocessing. **a** PET/CT delivered a low-dose CT (intrasystemly CT-derived *μ*-map not shown), and the resulting PET_PETCT_ served as standard of truth for the visual analysis in this study. **b** PET/MRI was acquired on the same day. Using different *μ*-maps but the same reconstruction parameters, four PET datasets were reconstructed in PET/MRI. Dixon-standard Dixon *μ*-map; CT-derived—Hounsfield units from the low-dose CT were bilinearly transferred to *μ* values which replaced values in the original Dixon *μ*-map; PseudoCT—a pseudo CT was calculated using a T1-weighted native MRI according to Poynton et al.[11]; BoneDixon-bone voxels *μ* values from the vendor specific ultrashort echo time (UTE) sequence were replaced in the original Dixon *μ*-map. **c** PET_CTderived_ served as standard for the quantitative analyses across different PET/MRI reconstructions in this study. For each patient, the percent deviations between and PET_Dixon/PseudoCT/BoneDixon_ and PET_CTderived_ were calculated, resulting in relatively small deviations compared to gold standard PET_CTderived_ if bone was accounted for (*top*) and larger deviations if bone was ignored (*bottom*)

PET/CT was performed on a Biograph 16 (Siemens Healthcare, Erlangen, Germany). Low-dose CT data for each patient were obtained (120 kVp, 41 mAs, 512 × 512 matrix, 55 slices, voxel size 0.59 × 0.59 × 3 mm^3^). Brain PET data were acquired in 3D mode over 5 min in one bed position, processed using standard correction methods, and reconstructed into a 256 × 256 matrix with 55 slices (voxel size 1.3 × 1.3 × 3.0 mm^3^) using 3D OSEM with four iterations, eight subsets, and a 5-mm Gaussian filter, resulting in PET_PETCT_ for each patient. Due to different scanner properties and reconstruction parameters, PET_PETCT_ was not quantitatively compared to any of the PET reconstructions from the PET/MRI (PET_Dixon/CTderived/PseudoCT/BoneDixon_). For the same reason, we reduced the scan time to the lowest possible of 5 min in PET/CT [9] as opposed to 20 min in PET/MRI. Figure 1 gives an overview of the image postprocessing steps and the different PET data obtained for comparative analysis.

### PET data postprocessing

(1) The PET data from PET/CT for each subject were co-registered to the PET data from PET/MRI, using six degrees of freedom (FLIRT, FSL ToolBox). The resulting transformation was also used to co-register the CT to the structural T1 MPRAGE MRI. Successful coregistration was visually verified and manually corrected if necessary using PMOD (PMOD 3.4, Zurich, Switzerland). An MPRAGE-based head mask (brain extraction tool, FSL ToolBox) was then superimposed on to the co-registered CT to automatically remove signals from extra cranial structures such as the CT eye shields and the CT patient table. CT-Hounsfield units were linearly transferred to *μ* values using a bilinear transfer function as described before [10]. The resulting *μ*-maps of the skull and brain were used to replace voxels in the original Dixon *μ*-maps (*CTderived μ-map*). (2) Using a recently introduced classifier [11], attenuation values for the head (including bone) were predicted from the anatomical information from the T1 MPRAGE (*PseudoCT μ-map*). (3) As a very simple alternative to the latter, these sophisticated algorithms, a *μ*-map was generated from the Dixon and UTE data (if available) imitating the UTE triple-echo (UTILE) method [4]. The bone information was extracted from UTE-based *μ*-maps and was laid over the original Dixon images using simple algebraic tools (miconv, micalc) from the ODIN framework (*BoneDixon μ*-*map*) [12]. Subsequently, all the resulting manipulated *μ*-maps were backtransfered to the console and used for reconstruction of PET_CTderived/PseudoCT/BoneDixon_ with exactly the same reconstruction parameters as outlined above (see Fig. 1).

### Visual PET image analysis

All PET datasets (PET_Dixon/CTderived/PseudoCT/BoneDixon_ and PET_PETCT_ for each patient) were visually evaluated in random order by three readers who were experienced in FDG brain PET data analysis and blinded to the patient details and diagnosis. For that purpose, the readers evaluated (1) transaxial PET slices of the FDG images, (2) three-dimensional z-score surface projections as obtained by the NEUROSTAT software [13], and (3) transaxial z-score slices as obtained by the Hermes BRASS software (Hermes Medical Solutions, Stockholm, Sweden). Overall, 12 brain areas were classified in a binary fashion as either hypometabolic or normal (frontal lobe right/left (r/l), temporal lobe r/l, parietal lobe r/l, occipital lobe r/l, anterior cingulate gyrus, posterior cingulate gyrus, cerebellum r/l). Moreover, the visual evaluation included a pattern analysis of the hypometabolism, and readers had to formulate a suspected diagnosis.

### Quantitative PET image analysis

Statistical parametric mapping (SPM8; Wellcome Trust Centre for Neuroimaging, London, UK) was used to compare the PET_CTderived_ datasets (which were considered the gold standard) with corresponding PET_Dixon/PseudoCT/BoneDixon_ datasets and to identify brain areas with divergent PET activity. For this purpose, spatial normalization was determined based on the co-registered T1-images and smoothing was performed with an 8-mm full-width at half-maximum on a Gaussian filter. A paired *t* test was applied for group comparison. Thresholds were set at *p* < 0.001, uncorrected. Further, to quantify AC-related regional differences between PET_CTderived_ and the corresponding PET_Dixon/PseudoCT/BoneDixon_ datasets, an atlas-based VOI analysis was performed by employing the HAMMERS template in PMOD. The percentage deviation of PET_Dixon/PseudoCT/BoneDixon_ as compared to PET_CTderived_ was calculated for each voxel across patients as follows:

$$ \left[\%\right]\ {\mathrm{Voxel}}_{\mathrm{Dixon}/\mathrm{PseudoCT}/\mathrm{BoneDixon}} = \left({\mathrm{PET}}_{\mathrm{Dixon}/\mathrm{PseudoCT}/\mathrm{BoneDixon}} - {\mathrm{PET}}_{\mathrm{CTderived}}\right)/{\mathrm{PET}}_{\mathrm{CTderived}}*\ 100 $$

Mean images were subsequently averaged across patients to obtain one mean-average PET image for each reconstruction.

## Results

### VOI analysis

The volumes of interest (VOI)-based quantificational analysis revealed that, across all VOIs, there was a −11.9, +2.3, and −7.4 % deviation of FDG uptake in PET_Dixon_, PET_PseudoCT_, and PET_BoneDixon_ compared to PET_CTderived_. Detailed results for the single brain areas are provided in Table 1. The highest uptake differences between PET_CTderived_ and PET_Dixon_ in favor of PET_CTderived_ were found in the frontal, parietal, and occipital lobes, and in the cerebellum (~13 to 16 %), while the lowest differences were found in deeper structures like the corpus callosum and the ventricles (~2 %). Similarly, the highest difference between PET_BoneDixon_ and PET_CTderived_ were detected in the frontal, temporal, and occipital lobe, and in the cerebellum (~8 to 10 %) and the lowest were found in the ventricles and the corpus callosum (~0 to 2 %). In contrast, the uptake differences between PET_CTderived_ and PET_PseudoCT_ were much lower and generally in favor of PET_PseudoCT_. In the temporal, frontal, and occipital lobe, and in the cerebellum, differences of up to ~3 to 4 % were found, whereas the difference in deeper brain structures was negligible (~0 to 2 % basal ganglia, brainstem, and corpus callosum).

### Visual analysis

**Fig. 2:**
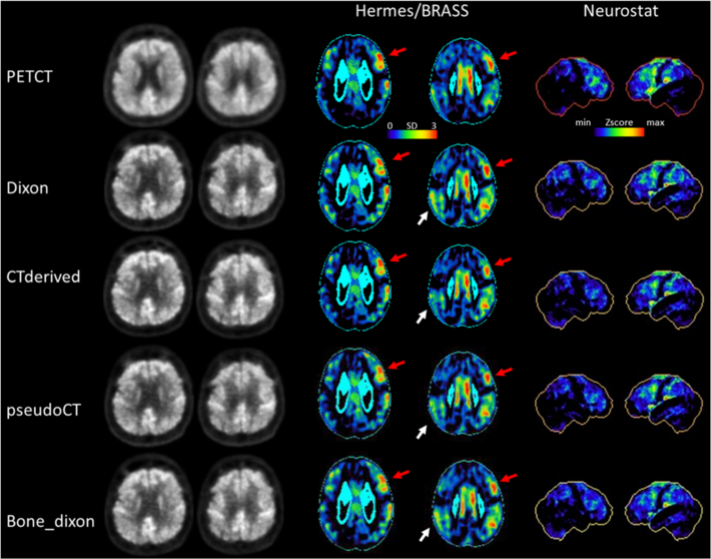
Patient example. Sixty-five-year-old women with clinical and imaging-based diagnosis of corticobasal degeneration. Relative FDG uptake was severely impaired in the left frontal regions (*red arrows*). Additionally, there was some degree of relative bilateral uptake deficiency in parietal areas which was more pronounced in PET_Dixon_ but also apparent in PET_BoneDixon_ as compared to PET_CTderived_ (*white arrows*), resulting in an imaging diagnosis of Alzheimer’s disease in PET_PseudoCT/BoneDixon_ for reader 1 and in PET_Dixon/PseudoCT/BoneDixon_ for reader 2. Note: readers were blinded to the clinical diagnosis

Using PET_PETCT_ as reference, in nine patients, the hypometabolism was either predominantly unremarkable or could not be related to any characteristic pathologic pattern so that the suspected diagnosis remained unspecific. In three patients, an Alzheimer’s dementia (AD) and in one patient a frontotemporal lobar degeneration (FTLD) was suspected. In one patient, no coherent suspected diagnosis could be formulated, and there was a mismatch compared to PET_PETCT_ according to the majority decision (Table 2, Fig. 2). This 65-year-old female patient suffered from a progressive movement disorder with spasticity in all four limbs, ataxia, and a cerebellar syndrome—the clinical and imaging-based diagnosis was corticobasal degeneration. Without knowledge on clinical symptoms, the blinded readers suspected this PET scan as either unspecific or considered an AD or a type of FTLD as the most likely diagnosis. However, no systematic reading error across the readers and the different PET reconstructions could be observed in this case. Also, using PET_PETCT_ as reference, in 85–100 % of patients, the suspected diagnosis from PET_Dixon/CTderived/PseudoCT/BoneDixon_ was correct for all readers, whereas none of the reconstructions were inferior to the others.

**Table 2:**
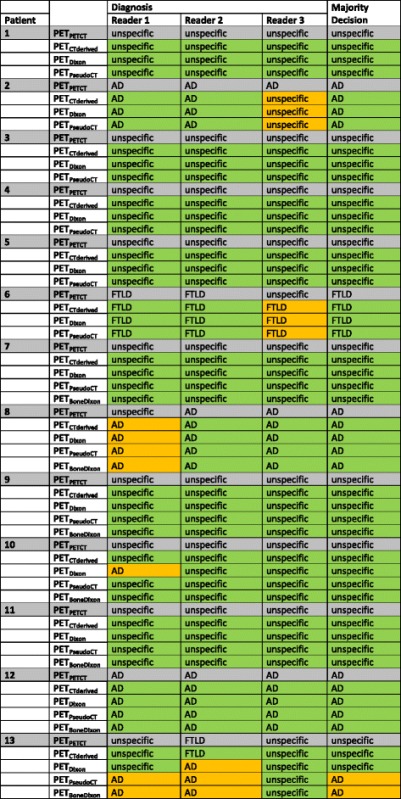
Suspected diagnosis in comparison to PET_PETCT_ gold standard

The suspected diagnosis from PET_CTderived_, PET_Dixon_, PET_PseudoCT_ and PET_BoneDixon_ did either match (*green*) or mismatch (*orange*) with the diagnosis from the gold standard PET_PETCT_. According to a majority decision, there was a mismatch only in patient 13 for PET_PseudoCT_ and PET_BoneDixon_. Note: despite the quantificational difference, the suspected diagnosis for this particular patient did not differ between PET_PETCT_ and PET_CTderived_ according to either of the readers. *AD* Alzheimer’s dementia, *FTLD* frontotemporal lobar degeneration

The number of hypometabolic regions per patient, as visually assessed, did not differ significantly between PET_CTderived_, PET_Dixon_, PET_PseudoCT_, and PET_BoneDixon_ in all readers and ranged from 1 to 2, 1 to 3, and 2 to 3 for readers 1, 2, and 3 (Table 3). Moreover, for PET_CTderived_, PET_Dixon_, PET_PseudoCT_, and PET_BoneDixon_, the number of correctly classified brain regions was determined according to the standard of truth PET_PETCT_. When interpreting Hermes BRASS from PET_Dixon_ as compared to PET_PseudoCT_, reader 2 classified significantly more brain regions correctly (*p* = 0.012). Apart from that, the number of correctly classified brain regions did not differ between PET_CTderived_, PET_Dixon_, PET_PseudoCT_, and PET_BoneDixon_ across patients, and no systematic inferiority of any reconstruction was observed. Numbers of correctly classified brain regions ranged from 9 to 11, 10 to 12, and 9 to 11 for readers 1, 2, and 3 (Table 3).

**Table 3:** Reading results

Reader		PET_PET/CT_	PET_CTderived_	PET_Dixon_	PET_PseudoCT_	PET_BoneDixon_
1	Hypometabolic areas (visual)	1.1 ± 1.7	1.3 ± 2.1	1.6 ± 2.0	1.3 ± 1.7	1.7 ± 1.7
	Hypometabolic areas (NEUROSTAT)	1.5 ± 2.1	1.8 ± 2.0	2.4 ± 2.1	2.3 ± 2.3	1.9 ± 2.0
	Hypometabolic areas (BRASS)	1.1 ± 1.6	1.9 ± 2.6	2.2 ± 2.3	2.1 ± 2.1	2.1 ± 2.1
	Correctly classified brain regions compared to PET_PET/CT_ (visual)		10.8 ± 1.7	10.6 ± 1.5	10.5 ± 1.9	9.1 ± 2.3
	Correctly classified brain regions compared to PET_PET/CT_ (NEUROSTAT)		10.9 ± 1.7	10.6 ± 1.8	10.7 ± 2	9.6 ± 2.5
	Correctly classified brain regions compared to PET_PET/CT_ (BRASS)		10.9 ± 1.7	10.5 ± 1.7	10.7 ± 1.7	9.6 ± 1.7
	Correct imaging diagnosis compared to PET_PET/CT_		11/13	11/13	11/13	7/7
2	Hypometabolic areas (visual)	1.4 ± 2.0	1.4 ± 1.9	1.3 ± 1.7	1.5 ± 2.2	1.7 ± 2.1
	Hypometabolic areas (NEUROSTAT)	2.8 ± 3.1	2.1 ± 2.4	2.5 ± 2.8	1.8 ± 2.3	3.0 ± 2.7
	Hypometabolic areas (BRASS)	2.2 ± 2.9	1.9 ± 2.4	2.2 ± 2.8	1.1 ± 2.0	1.3 ± 2.1
	Correctly classified brain regions compared to PET_PET/CT_ (visual)		11.5 ± 0.7	11.6 ± 0.9	10.8 ± 1.8	11.1 ± 1.1
	Correctly classified brain regions compared to PET_PET/CT_ (NEUROSTAT)		11.2 ± 1.1	11.2 ± 1.4	10.4 ± 2.4	10.7 ± 1.7
	Correctly classified brain regions compared to PET_PET/CT_ (BRASS)		11.0 ± 1.2	11.4 ± 0.7*	10.4 ± 1.5*	10.1 ± 1.5
	Correct imaging diagnosis compared to PET_PET/CT_		13/13	12/13	12/13	6/7
3	Hypometabolic areas (visual)	1.8 ± 2.5	2.1 ± 1.8	2.6 ± 1.9	2.5 ± 1.9	2.7 ± 1.9
	Hypometabolic areas (NEUROSTAT)	2.2 ± 2.5	1.9 ± 1.6	2.5 ± 1.9	2.5 ± 1.8	2.1 ± 1.6
	Hypometabolic areas (BRASS)	2.2 ± 2.5	1.9 ± 1.7	2.4 ± 1.7	2.6 ± 1.9	2.1 ± 1.6
	Correctly classified brain regions compared to PET_PET/CT_ (visual)		10.8 ± 1.7	10.6 ± 1.5	10.5 ± 1.9	9.1 ± 2.3
	Correctly classified brain regions compared to PET_PET/CT_ (NEUROSTAT)		10.7 ± 1.6	10.8 ± 1.3	10.5 ± 1.9	9.1 ± 2.3
	Correctly classified brain regions compared to PET_PET/CT_ (BRASS)		10.9 ± 1.7	10.6 ± 2.8	10.7 ± 2.0	9.6 ± 2.5
	Correct imaging diagnosis compared to PET_PET/CT_		11/13	11/13	11/13	7/7

PET_PET/CT_, PET_CTderived_, PET_Dixon_, PET_PseudoCT_, and PET_BoneDixon_ from 13 patients were evaluated by three experienced readers. For each PET, 12 brain areas were classified as either hypometabolic or normal according to the visual impression and according to the semiquantitiative approaches NEUROSTAT and BRASS. For PET_CTderived_, PET_Dixon_, PET_PseudoCT_, and PET_BoneDixon_, the number of correctly classified brain regions was determined according to the standard of truth PET_PETCT_. Moreover, the readers were asked to formulate an imaging diagnosis on the basis of the PET data without clinical information. The imaging diagnosis was also compared to the standard of truth PET_PETCT_. For PET_PETCT_, PET_CTderived_, PET_Dixon_, and PET_PseudoCT_ (PET_Bonedixon_), paired (two-sampled) *t* tests (two-sided; *α* = 0.05) were calculated. *α* was Bonferroni corrected for multiple comparisons. The number of hypometabolic areas across the patients did not differ significantly between PET_CTderived_, PET_Dixon_, PET_PseudoCT_, and PET_BoneDixon_ and the reference: PET_PETCT_ in either of the readers. *Reader 2 classified significantly more brain regions correctly using BRASS from PET_Dixon_ as compared to PET_PseudoCT_ (*p* = 0.012). Apart from that, the number of correctly classified brain regions did not differ between PET_CTderived_, PET_Dixon_, PET_PseudoCT_, and PET_BoneDixon_ across patients. No systematic inferiority of any reconstruction was observed

### Statistical parametric mapping (SPM) analysis

**Fig. 3:**
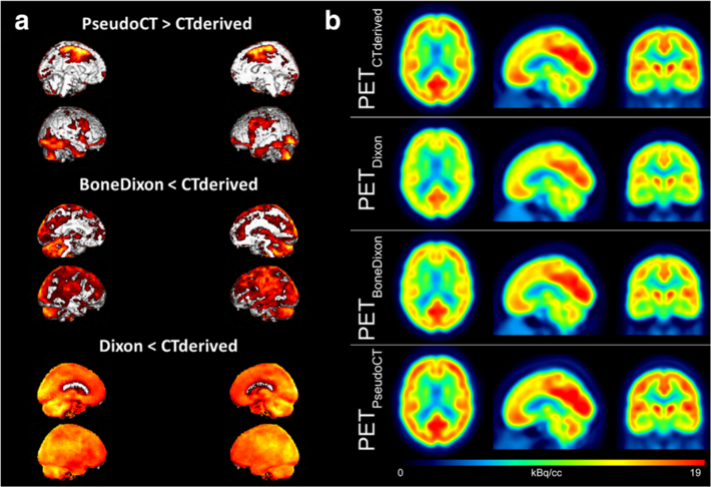
Comparison of different attenuation correction approaches in FDG brain PET/MRI. **a** Relative FDG uptake comparison by statistical parametric mapping between PET_PseudoCT/BoneDixon/Dixon_ vs. PET_CTderived_ from the 13 patients investigated (note: PET_BoneDixon_ was available in seven patients only). Midsagittal and lateral projections: significance level of *p* < 0.001; *T* value >3.9 (*T* > 5.2 for PET_BoneDixon_ due to lower sample size). Relative FDG uptake was significantly lower in PET_Dixon/BoneDixon_ as compared to PET_CTderived_ in a widespread area covering occipital, parietotemporal, and parieto-occipital cortices as well as the cerebellum. FDG uptake was significantly higher in PET_PseudoCT_ as compared to PET_CTderived_ in more restricted areas, including the primary and parietotemporal cortices as well as the cerebellum. **b** Mean images across all patients for the different attenuation correction approaches (after spatial normalization)

The relative FDG uptake was significantly lower in PET_Dixon_ as compared to the reference PET_CTderived_ in a widespread area covering the whole cortex and the cerebellum (Fig. 3a). The effect was less pronounced in PET_BoneDixon_ in terms of intensity and level of significance but still affected the whole brain. Relative FDG uptake was significantly higher in PET_PseudoCT_ as compared to PET_CTderived_ in more restricted areas, including the primary and parietotemporal cortices as well as the cerebellum.

## Discussion

In this sequential brain FDG PET/MRI and PET/CT study in 13 patients, the Dixon *μ*-map was systematically complemented by bone information from (1) CT as obtained by PET/CT resulting in PET_CTderived_, by (2) MR anatomy information resulting in PET_PseudoCT_, and (3) by help of an UTE sequence resulting in PET_BoneDixon_. We found that ignoring bone in this patient population did result in ~12 % uptake underestimation. This is in line with prior studies stating that due to the omission of bone, the underestimation of the PET signal ranges from to 11 to 25 % [3–5]. When using an ultrashort echo sequence for bone classification or accounting for cortical bone by use of a recently proposed [11] MR-based method to predict continuous attenuation values for the bony skull, the FDG uptake estimation was improved (~7 % underestimated or ~2 % overestimated). Also, these results are in line with prior studies [11, 14]. However, to the best of our knowledge, the only FDG PET study evaluating the clinical impact of different attenuation correction approaches suffered from different reconstruction parameters and scanner properties [8]. In our study, the only source of variation between the PET datasets were the *μ*-maps themselves; thus, the data was directly comparable without normalization. This allowed to study the direct clinical impact of the above-described AC-related FDG uptake presentation differences on the clinical FDG brain PET diagnosis. Here, even though the unaffected cortical PET activity in PET_Dixon/BoneDixon_ was substantially lower, the clinical impact was neglibile; three experienced readers did not rate systematically more brain areas as “hypometabolic” after visual inspection of the PET_Dixon/CTderived/PseudoCT/BoneDixon_ slices and after evaluation of the statistical analyses of the normalized PET data (NEUROSTAT and BRASS). In line with that, a clinical evaluation of different ACs in comparison to the gold standard resulted in no differences in the number of hypometabolic areas as identified by the readers for each patient. Furthermore, the FDG PET diagnosis was not more severe in AC approaches that tend to underestimate the cortical PET signal (PET_BoneDixon_, PET_Dixon_) in any of the patients; despite the above mentioned quantificational difference, the large proportion of unspecific cases was not misdiagnosed as pathologic in PET_BoneDixon_, and PET_Dixon_ as compared to PET_PETCT_, PET_CTderived_, and PET_BoneDixon_. Taken together, the quantificational difference by the omission of bone did not translate into a systematic diagnostic error in our FDG PET/MRI(CT) study. This was probably because this global effect may change intensities of apparent cortical hypometabolism but not the overall pattern of hypometabolism, which is decisive. Moreover, this global effect did not lead to the typical decrease-vs.-normal contrast along the gray matter (e.g., the occipital cortex exhibits normal glucose metabolism and the adjacent parietal cortex does not in Alzheimer’s dementia).

A limitation of this investigation is the limited number of subjects examined that does not represent the larger variety of diseases whose diagnosis is often supported by FDG brain PET (namely, Alzheimer’s disease, frontotemporal dementia, dementia with Lewy bodies, Parkinson’s disease, progressive supranuclear palsy, multiple system atrophy, corticobasal degeneration, and Huntington’s disease). As a matter of future research, the evaluation of the impact of AC-related FDG PET quantificational differences on the evaluation of patients with atypical parkinsonian syndrome, like multiple system atrophy or supranuclear palsy, would be interesting. In these patients, subcortical regions as well as the cerebellum which is heavily surrounded by bony structure might also be involved, and the AC-related quantificational error could thus be of clinical relevance.

Moreover, (at least) for research applications, an MR-AC-related error of ~12 % for FDG brain PET in case of bone omission needs to be considered and should be further decreased by the usage of recently introduced MR-based algorithms to predict continuous *μ* values. However, the difference in FDG uptake of 2.3 % between PET_PseudoCT_ and PET_CTderived_ as observed in this study seems acceptable for most brain regions even for research applications.

Of note, the relative robustness of diagnostic accuracy against AC-related errors as observed in this present FDG brain PET study cannot simply be translated to other PET tracers or to other body regions without further investigation. Even though a recent study observed a similarly limited impact of the Dixon based MR-AC on clinical diagnosis in amyloid-PET, we would be careful in assuming that this holds true for neurological PET studies in general [15]. The contrast between the cortical signal (on the surface) and the white matter signal (deeper location) may compromise the quantification of cerebral blood flow [16] and may have an impact on the quantification of amino acid turnover in small tumor lesions close to the skull [7]. Thus, a standardized vendor-based implementation of advanced AC algorithms that can provide accurate skull CT surrogates [11, 17] remains highly desirable for future PET/MRI systems and their clinical and research applications.

## Conclusions

Despite a quantitative error by the omission of bone in MR-AC, clinical quality of brain [18 F]FDG is not relevantly affected in this patient cohort with suspected dementia and movement disorders. Thus, brain [18 F]FDG PET can already, even now with suboptimal MR-AC, be utilized for clinical routine purposes. Advanced AC algorithms that can provide accurate skull CT surrogates reduced the difference in FDG uptake to a minimum that is even acceptable for quantification in research applications.

### Ethics approval and consent to participate

All retrospective analyses involving human participants in the present study were approved by the local institutional Review Board (# 065-14-10032014) and were in accordance with the principles of the 1964 Declaration of Helsinki and its later amendments or comparable ethical standards. Informed consent was obtained.
